# Direct healthcare burden and costs of operatively managed prosthetic joint infection in a public hospital setting

**DOI:** 10.5194/jbji-11-667-2026

**Published:** 2026-11-04

**Authors:** Matthew Condon, Malak Alwaheed, Jillian O'Carroll, Alan Molloy

**Affiliations:** 1 Trauma and Orthopaedics Department, St Vincent's University Hospital, Dublin, Ireland; 2 University College Dublin School of Medicine, Dublin, Ireland

## Abstract

Prosthetic joint infection (PJI) following arthroplasty is associated with high morbidity and financial cost. Patients often undergo multiple complex procedures, prolonged inpatient stays and outpatient parenteral antibiotic therapy (OPAT). We aim to quantify the economic implications of the inpatient and OPAT costs of the management of private patients treated in the public healthcare system. Retrospective analysis of operative PJI cases between September 2020 and September 2025 was performed. Included patients were those who had primary total hip replacement (THR) or total knee replacement (TKR) for osteoarthritis or trauma. Data included inpatient stay, OPAT utilization, surgical procedures (debridement, antibiotics, and implant retention (DAIR); and revisions), and private/public status at index procedure. Costs were estimated using available Health Service Executive (HSE) information on operative costs and bed days. There were 40 operative PJI cases in our centre over the study period. There were 22 TKRs and 18 THRs. Eighteen had their initial surgery publicly, whereas 14 had their initial surgery privately. A total of 19 patients underwent DAIR while the rest underwent one or several revisions. Combined total length of stay was 1796 bed days. The operative PJI burden over that period was EUR 5 005 339 (median EUR 84 232 per episode) or approximately EUR 1 000 000 yr^−1^. Fourteen private patients treated in our centre had publicly funded treatment at an estimated cost of EUR 1 967 270 over a 5-year period or EUR 393 454 yr^−1^. Operative PJI imposes a substantial clinical and financial burden. Private patients treated in public hospitals contribute to the total costs, raising the justification for private healthcare contributions to publicly treated complications arising after private procedures.

## 1 Introduction

Prosthetic joint infection (PJI) is an uncommon but major complication of primary total joint arthroplasty, with reported incidence rates of approximately 1 % (Springer et al., 2017). Infection can be a life-changing outcome for patients, often requiring revision surgery, prolonged antibiotic therapy, and lengthy hospital admissions. The management of these cases is associated with a high financial cost and burden on hospital resources, despite the increasing use of outpatient parenteral antibiotic therapy (OPAT) which has been shown to significantly reduce costs associated with inpatient bed days (Reidy et al., 2025). Several private arthroplasty centres exist in Dublin, providing primary arthroplasty services. However, they are not equipped to perform revision surgery or to accommodate the potentially prolonged hospital admissions required for the management of PJI. Patients in our hospital's catchment area needing assessment or intervention for PJI present to the 24 h emergency department at our trauma unit, regardless of the public/private nature of their primary arthroplasty. The subsequent care received is funded by the public healthcare system.

In this study, we aim to quantify the economic burden of PJI on our hospital resources and determine what proportion of the costs originates from complications of operations performed in the private sector. We discuss the further effects of these cases on our centre and the public healthcare system, as well as possible solutions.

## 2 Methods

This is a retrospective analysis of operative PJI cases between September 2020 and September 2025 at our trauma unit. Included patients were those who had a documented history of primary total hip replacement (THR) or total knee replacement (TKR) for osteoarthritis or trauma. Primary arthroplasty for native joint septic arthritis was excluded. An operative PJI case was defined as any patient who underwent one or several operations for suspected or confirmed PJI of a TKR or THR, with EBJIS (McNally et al., 2021) criteria applied and consultant decision-making to support “infection likely” cases. A case was defined as an individual patient, regardless of number of episodes, operations, or overall cost. The cost calculation was isolated to the costs incurred during the study period alone.

Data collected included inpatient stay, OPAT utilization, surgical procedures (debridement, antibiotics, and implant retention (DAIR); and revisions), and private/public status at index procedure. Costs were estimated using available HSE information on operative costs and bed days. A recent HSE official estimate for the average cost of 1 bed day in a public hospital is EUR 1204 (Health Service Executive, 2025a). The HSE Admitted Patient Price List (Health Service Executive, 2024) is published annually and was used to estimate surgical costs for the procedures undergone by our cohort. The operative costs are detailed in Table 1. DAIR was assigned “revision surgery of intermediate complexity”, whereas revisions were assigned “revision surgery of major complexity”. Although there is an annual price variation, publicly available price lists are published for 2024 and 2025 (Health Service Executive, 2024, 2025b). The difference in cost for I32A, the code chosen for TKR revision, is of a EUR 499 or a 1.25 % increase. For I31A, this difference is EUR 100 or 0.16 %. These changes were deemed minimal enough to estimate costs through the study period using the 2024 price list. Historical expenditures were not inflation adjusted.

**Table 1:** Estimated costs for PJI-related procedures according to the HSE Admitted Patient Price List.

Procedure	Cost (EUR)	Procedure code
Joint washout	5483	I24B
DAIR (revision of intermediate complexity)	26 106	I12B
TKR revision (revision of major complexity)	40 216	I32A
THR revision (revision of major complexity)	60 956	I31A

Although initially the cost of oral tablets was considered, the costs were found to be insignificant compared to other costs. As an example, as per the primary Care Reimbursement Service website, the cost of 20 years of 1 g amoxicillin BD is EUR 2277.60. The cost of doxycycline 100 mg BD for 10 years is EUR 1812 (Health Service Executive, 2026). These costs are also covered to a varying degree by private prescription charges rather than public hospital resources.

The daily cost of OPAT was conservatively estimated at around EUR 100 d^−1^, based on information acquired from the Irish OPAT control centre and European studies (Reidy et al., 2022; Scottish Health Technologies Group, 2023; Chapman et al., 2009; González-Ramallo et al., 2017). Total costs per patient were therefore estimated using the following equation:

$$\begin{array}{cc} \text{Total costs} & =\text{surgical procedure costs}+\\ & \text{total inpatient bed-day costs}+\text{total OPAT costs} \end{array}$$

Table 2 details the cost calculation of two typical cases.

**Table 2:** Sample cost calculation for two example patients in our cohort.

	Procedure(s) (cost)	Bed days (cost)	OPAT days (cost)	Total costs
Patient 1	DAIR (EUR 26 106)	9 (EUR 10 836)	42 (EUR 4200)	EUR 41 142
Patient 2	Two-stage revision TKR (EUR 60 956 × 2 = EUR 121 912)	19+5=24 (EUR 28 896)	42 (EUR 4200)	EUR 155 008

## 3 Results

There were 40 operative PJI cases in our centre over the study period, of which 22 patients were post-TKR and 18 post-THR. The median age was 72. There were 21 females and 19 males.

### 3.1 Setting of primary arthroplasty

Eighteen had their initial surgery publicly, while 14 had their initial surgery privately.

**Table 3:** Setting of primary arthroplasty.

Setting	Count, %
Public	N=18 , 45 %
Private	N=14 , 35 %
External	N=1 , 2.5 %
Unknown	N=7 , 17.5 %
Total	N = 40, 100 %

### 3.2 Timing of diagnosis

Table 4 describes the costs associated with PJI in our cohort, subdividing PJI timing from index procedure and public/private distinction. Most (12 public, nine private) patients were diagnosed as late PJIs and account for a majority of the costs (EUR 2 908 340). Five patients in the private cohort were diagnosed with acute or delayed PJI, to a cost of EUR 474 868 over the study period.

**Table 4:** Cost breakdown by public/private status and PJI classification.

	Number of public cases (cost)	Number of private cases (cost)	Total cases (cost)
Acute (within 3 months)	N = 2 (EUR 359 894)	N = 3 (EUR 271 102)	N = 5 (EUR 630 996)
Delayed (3 months to 2 years)	N = 4 (EUR 317 522)	N = 2 (EUR 203 766)	N = 6 (EUR 521 288)
Late (over 2 years)	N = 12 (EUR 1 415 938)	N = 9 (EUR 1 492 402)	N = 21 (EUR 2 908 340)
Total	N = 18, 56.25 % (EUR 2 093 354)	N = 14, 43.75 % (EUR 1 967 270)	N = 32 (external and unknown excluded)
			(EUR 4 060 624)

### 3.3 Type of surgery

Operations that our patients underwent are detailed in Table 5. It is worth noting this does not represent what operations were successful in resolving a PJI as some patients were still undergoing follow-up outside of the study period. Nineteen patients underwent DAIR, and the rest underwent one or several revisions.

**Table 5:** Operations by type.

Operation(s)	Count, %
DAIR only	N = 19, 47.5 %
Multiple revisions/DAIR	N = 7, 17.5 %
Single-stage revision only	N = 1, 2.5 %
Two-stage revision only	N = 11, 27.5 %
First-stage revision (part of a two-stage) only	N = 2, 5 %

### 3.4 Operative, bed day, and OPAT costs

The combined total length of stay was 1796 bed days for a median of 21 d. The total number of OPAT days was 1855 or a median of 42 d (6 weeks). The total costs are detailed in Table 6.

**Table 6:** Breakdown of total and median costs over the study period.

	Total (EUR)	Median (EUR)
Surgical	2 657 455	48 955
Bed days	2 162 384	25 284
OPAT	185 500	4200
Overall	5 005 339 or 1 001 068 annually	84 232

The total estimated direct hospital and OPAT costs associated with operative THR/TKR PJIs in our centre over the 5 year study period was approximately EUR 5 million or EUR 1 million annually. The median cost per patient was EUR 84 232.

There was variation in the cost per case. Cost per case ranged from EUR 32 624 (day admission and transfer to private facility post-operatively, single DAIR procedure, and 6 weeks OPAT) to EUR 744 044 (405 d admission, three revision surgeries and Girdlestone procedure, and 18 weeks OPAT). The portion of these costs represented by patients who underwent primary Total joint arthroplasty (TJA) in a private hospital was EUR 1 967 270 or EUR 393 454 annually. There was no difference between the costs of this cohort and the cohort of those who had their primary TJAs performed in a public hospital ( p=0 .747).

The mean number of operations was 1.64 in the private cohort and 1.50 in the public cohort, and this difference was insignificant ( p=0 .897).

The median cost of private patients was EUR 67 996, and the median cost of public patients was EUR 84 232. There was no significant difference between the cohorts in overall costs ( p=0 .633).

The median length of stay was 22 d for private patients and 21 d for public patients. This difference was not significant ( p=0 .633). Even removing from the calculations those patients who were transferred to a private facility post-operatively (cutting short their length of stay), the difference was still insignificant ( p=0 .322).

Seven patients were not distributed into the public/private cohorts because the origin of their primary arthroplasty could not be confidently identified. The total costs attributable to these patients are EUR 816 195 or a median EUR 87 040. This is compared to total costs of EUR 4 189 144 or a median of EUR 82 628 in the rest of the cohort. These seven unknown cases mean the actual cost attributable to the “private” cohort (if all “unknown” cases are added) may be up to EUR 2 783 465 or over half the total costs in the study period.

## 4 Discussion

The primary goal of this study was to estimate the costs to a trauma unit of running a service equipped to manage PJIs. This is a complex, often comorbid cohort requiring intense resource allocation (Migliorini et al., 2026; Alt et al., 2025). At an individual level, there is a significant variation in the cost of treating a PJI, due to recurrences, the potential for repeat revisions, and variations in the required length of hospital stay and intensity of OPAT. In addition to the costs estimated above, there are costs associated with outpatient clinics and follow-up investigations, which were outside of the scope of this study. Indirect costs also arise from patients being out of the workforce for the duration of their treatment, which is difficult to estimate but likely significant to both the patients themselves and wider society.

It is important to underscore that although OPAT has running costs, it is a successful and essential cost-saving initiative, estimated to save the HSE EUR 20 million annually in inpatient bed days (Reidy, 2022). OPAT represents a minority of the costs associated with PJI in our study, and our results should be seen as a testament to its cost-effectiveness.

### 4.1 Public and private cases

To address the two cohorts presented in the results, we found no statistically significant difference in the length of stay, number of operations, or costs associated with treating the “private” cohort compared with the “public” cohort. The contrast with a similar study in another centre is interesting. Clausen et al. (2026) found offsite primary TKA resulted in higher mean operative costs, longer length of stay, operations, and admissions. The possible explanations for this are effect size, differing decision-making priorities between centres and consultants, and differing cost estimation methods. 

### 4.2 Direct and indirect impacts of inpatient bed days

The financial implications of an approximate 360 inpatient bed days yr^−1^ is not limited to the cost of treatment. Although not assessed directly through this study, each occupied orthopaedic bed results in an increased likelihood of cancellations on the elective surgery list, which in a tertiary hospital often affects complex cases awaiting surgery that cannot be performed elsewhere. Therefore, the impact on surgical lists is not limited only to the days patients are undergoing revision surgery for PJI. Delays or cancellations in elective arthroplasty services may keep patients out of the productive workforce and increase the use of analgesia, outpatient appointments, and dissatisfaction with the health system. Theatre time being occupied with operations for PJI can also delay ambulatory trauma cases, where trauma should be the primary service provided in these centres. An NHS report from 2022 confirms the real downstream effects of hospital overcrowding on surgical lists, as 17.3 % of list cancellations were secondary to a lack of physical hospital capacity (Moonesinghe et al., 2022).

Hospital overcrowding has a real impact on the provision of services and is a prominent talking point in the Irish media, with particular attention paid to outpatient appointments and elective surgery waiting lists (Reporter, 2026; Bowers, 2026). A recent study from the UK demonstrates that delays to elective orthopaedic surgery results in excess personal costs and diminished health-related quality of life as measured by quality-adjusted life years (Scott et al., 2025).

Delays to elective surgery results in obvious negative effects at the individual level as well as broader economic costs. Recent research from Scotland shows that longer wait times for hip and knee arthroplasty results in an increased likelihood of pre-operative work cessation and correlates with post-operative failure to return to work. The economic impact of the difference between a 3-month wait and a 9-month wait for elective arthroplasty in this study was an additional GBP 44.5 million annually or around GBP 8000 per case. Evidently there is a significant benefit to the economy in reducing wait times for elective surgery, and cancellations secondary to emergency PJI cases will have a negative impact (Morrison et al., 2026).

### 4.3 Impact of different priorities

It is apparent that although the public health system needs to consider the accepted risk of PJI affecting patients post-elective arthroplasty and its financial implications, private institutions may encounter fewer of these costs due to PJI care being undertaken in public institutions, potentially resulting in asymmetric expenditures. Private institutions favour day case or short-stay operations for high patient turnover and case completion. Complex revisions associated with prolonged inpatient stays and the implications on further bed availability entail costs that disadvantage the private sector. The provision of intensive or high dependency care services may mean that certain cases require a bed in a tertiary centre, but many of our cases undergoing DAIR or single revisions could be performed in other centres, assuming adequate case selection. Resource allocation should account for the additional workload attached to treating PJI from both public and private sectors.

### 4.4 Possible solutions

Although PJIs are effectively managed in our centre, treating patients who had primary surgery performed externally poses a financial dilemma. Should private facilities be capable of treating prosthetic joint infections and performing revision surgery for a relatively small cohort, or, perhaps more simply, should the costs associated with treating complications of private arthroplasties be partially or wholly funded by private insurance companies? The answer cannot be black and white, particularly as we try to define direct complications. Most of our cohort was diagnosed as late PJI, as illustrated in Table 4. Approximately EUR 500 000 in costs is attributable to PJIs diagnosed either as acute or delayed. Late PJIs make up a majority of our cohort, both from public and private centres. Should a late PJI that arises 15 years post-arthroplasty be considered a complication of that arthroplasty? Although most would likely consider the late PJI as a separate episode of care to the primary arthroplasty, it may equally be overly simplistic to consider responsibility for the management of these to lie entirely outside the original institution.

Furthermore, the potential additional cost to insurers may be passed on to patients seeking private insurance and undergoing arthroplasty in a private centre. It is important for a solution not to result in a transfer of burden of primary arthroplasty to the public system due to a decrease in profitability but rather an equitable distribution of responsibility to maximize the output of both institutions, which can and should have different priorities.

An ideal system treats each patient in a centre that is not over-resourced in comparison to their needs in order to increase capacity for those requiring access to specialized care. A formalized referral system between public and private centres for case selection to assess the need for tertiary care would allow private centres the confidence of treating PJI while maintaining a safety net should case complexity necessitate transfer. Public elective orthopaedic hospitals refer to predetermined acute hospitals as needed through agreed-upon pathways. Conversely, unwell patients presenting acutely to public emergency departments following private elective operations should have an available route to be treated in the institution where the operation was performed, where this is appropriate.

### 4.5 Limitations

The costs are estimated using available documentation, and despite accounting for the major expenses in the care of PJIs, exact costs for each episode are not precisely calculated. Given the retrospective nature of the study, we were at times limited by documentation. As an example, we were unable to find the setting of the original arthroplasty in seven of our cohort.

The calculated total bed days directly due to PJI is potentially underestimated due to patients transferring to a private facility during their stay and potentially overestimated by the fact that there were admissions with other active diagnoses requiring management. 

## 5 Conclusions

Prosthetic joint infection imposes a substantial clinical and financial burden on the public healthcare system. Resources should be therefore allocated to centres performing revision surgery for infected arthroplasties originating from their cohort and external cohorts presenting for treatment in those centres.

Operative and bed-day costs make up a majority of the costs, with OPAT contributing despite its critical overall cost-saving nature. Private patients treated in public hospitals add to the total costs in a significant way and have knock-on effects on public elective and trauma lists. It is justifiable to debate where the funds to treat this cohort should be sourced. Possible solutions include cost-sharing initiatives between public and private systems or streamlined referral pathways between centres. It would be relevant to conduct similar reviews in other centres treating PJIs in Ireland, with a view to estimating a national cost of PJI and adequately funding initiatives that may help to reduce those costs.

## Data Availability

Anonymized data may be made available by the corresponding author on request, subject to approval by the local data governance body.
